# Aqua­[1-(1,10-phenanthrolin-2-yl-κ^2^ *N*,*N*′)-1*H*-pyrazol-3-amine-κ*N* ^2^](sulfato-κ*O*)copper(II) methanol monosolvate dihydrate

**DOI:** 10.1107/S1600536812014134

**Published:** 2012-04-13

**Authors:** Liang Yuan, Liu Shu Lian, Chi Yan Hui, Zhao Yan Xia, Shi Jing Min

**Affiliations:** a College of Chemistry, Chemical Engineering and Materials Science, Shandong Normal University, Jinan 250014, People’s Republic of China; b School for Cadres of Shandong Bureau of Quality and Technical Supervision, Jinan 250014, People’s Republic of China; c Shandong Academy of Medical Science Graduate Education Center, Jinan 250062, People’s Republic of China

## Abstract

In the title compound, [Cu(SO_4_)(C_15_H_11_N_5_)(H_2_O)]·CH_3_OH·2H_2_O, the Cu^II^ ion is in a distorted square-pyramidal geometry, in which three N atoms from the chelating 1-(1,10-phenanthrolin-2-yl)-1*H*-pyrazol-3-amine ligand and one O atom from a sulfate anion define the basal plane and the O atom from the coordinating water mol­ecule is located at the apex. In the crystal, hydrogen-bonding inter­actions involving the coordinating and solvent water mol­ecules, the methanol solvent mol­ecule and the amine group (one with an intra­molecular inter­action to one of the sulfate O atoms) of the complex are observed. π–π inter­actions between symmetry-related phenantroline moieties, with a shortest centroid–centroid inter­action of 3.573 (2)°, are also present.

## Related literature

For related structures, see: Li *et al.* (2011**a* ▶,b* ▶).
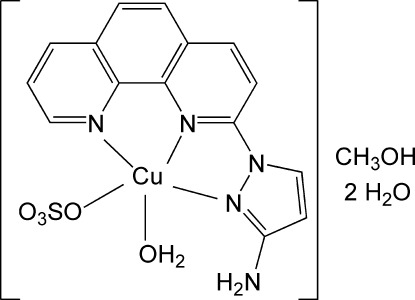


## Experimental

### 

#### Crystal data

[Cu(SO_4_)(C_15_H_11_N_5_)(H_2_O)]·CH_4_O·2H_2_O
*M* *_r_* = 506.98
Monoclinic, 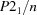

*a* = 8.0190 (13) Å
*b* = 18.489 (3) Å
*c* = 14.086 (2) Å
β = 104.551 (2)°
*V* = 2021.4 (6) Å^3^
*Z* = 4
Mo *K*α radiation
μ = 1.24 mm^−1^
*T* = 298 K
0.22 × 0.15 × 0.11 mm

#### Data collection

Bruker SMART APEX CCD diffractometer
Absorption correction: multi-scan (*SADABS*; Sheldrick, 1996 ▶) *T*_min_ = 0.772, *T*_max_ = 0.876
11702 measured reflections
4394 independent reflections
3434 reflections with *I* > 2σ(*I*)
*R*_int_ = 0.038

#### Refinement

*R*[*F* ^2^ > 2σ(*F* ^2^)] = 0.047
*wR*(*F* ^2^) = 0.119
*S* = 1.03
4394 reflections
281 parameters
H-atom parameters constrained
Δρ_max_ = 0.52 e Å^−3^
Δρ_min_ = −0.31 e Å^−3^

### 

Data collection: *SMART* (Bruker, 1997 ▶); cell refinement: *SAINT* (Bruker, 1997 ▶); data reduction: *SAINT* (Bruker, 200 ▶); program(s) used to solve structure: *SHELXTL* (Sheldrick, 2008 ▶); program(s) used to refine structure: *SHELXTL*; molecular graphics: *SHELXTL*; software used to prepare material for publication: *SHELXTL*.

## Supplementary Material

Crystal structure: contains datablock(s) I, global. DOI: 10.1107/S1600536812014134/wm2604sup1.cif

Structure factors: contains datablock(s) I. DOI: 10.1107/S1600536812014134/wm2604Isup2.hkl

Additional supplementary materials:  crystallographic information; 3D view; checkCIF report

## Figures and Tables

**Table 1:** Selected bond lengths (Å)

Cu1—O3	1.906 (2)
Cu1—N2	1.934 (2)
Cu1—N3	2.068 (2)
Cu1—N1	2.090 (2)
Cu1—O5	2.220 (2)

**Table 2:** Hydrogen-bond geometry (Å, °)

*D*—H⋯*A*	*D*—H	H⋯*A*	*D*⋯*A*	*D*—H⋯*A*
O5—H4⋯O7	0.89	1.79	2.671 (4)	167
O5—H5⋯O8	0.89	1.90	2.774 (4)	164
O6—H12⋯O1	0.85	1.89	2.668 (4)	152
O7—H6⋯O6	0.84	2.10	2.878 (5)	153
O7—H7⋯O4^i^	0.89	1.84	2.726 (4)	173
O8—H17⋯O6^ii^	0.90	2.07	2.906 (5)	154
O8—H18⋯O2^iii^	0.90	2.09	2.957 (4)	163
N5—H5*A*⋯O4	0.86	2.19	2.916 (4)	143
N5—H5*B*⋯O2^ii^	0.86	2.17	3.022 (4)	175

Symmetry codes: (i) 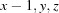; (ii) 
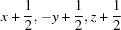
; (iii) 
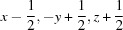
.

**Fig. 1.**
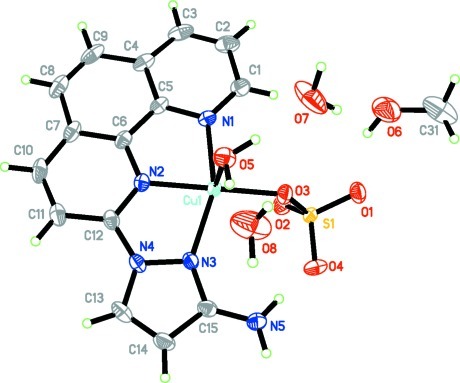
The asymmetric unit of the title compound with displacement ellipsoids shown at the 30% probability level.

**Fig. 2.**
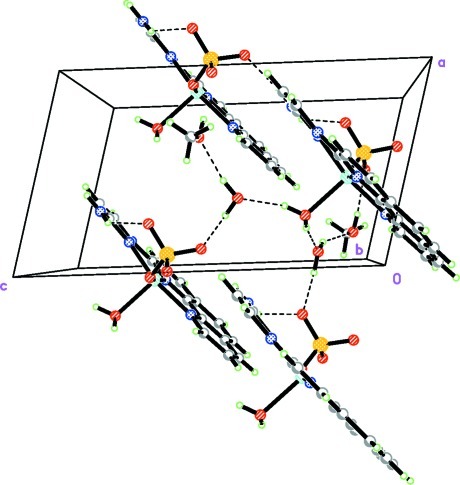
Unit cell of (I), showing the packing features consolidated by hydrogen bonding interactions

## supplementary crystallographic information

### Comment

Derivatives of 1,10-phenanthroline play an important role in coordination chemistry and many of such complexes have been reported with these types of derivatives as ligands, e.g. Li *et al.* (2011**a*,b*) for closely related Cu^II^ complexes. To the best of our knowledge, there is no report for a complex with 1-(1,10-phenanthrolin-9-yl)-1*H*-pyrazol-3-amine as ligand. Herein we report the crystal of the solvated title complex, (I).

The molecular structure of (I) is shown in Fig. 1. The Cu^II^ ion is is a distorted square-pyramidal coordination geometry with the O atom of the water molecule at the apex and three N atoms from the ligand and one O atoms from the sulfate anion in the basal plane. The non-hydrogen atoms from the 1-(1,10-phenanthrolin-9-yl)-1*H*-pyrazol-3-amine ligand make an approximate plane within 0.074 Å (rms deviation) with a maximum deviation of 0.145 (3) Å for the N5 atom.

In the crystal, the uncoordinated water molecules, the methanol molecule and the metal complex are connected to each other by hydrogen bonding interactions as shown in Table 2 and Figure 2. The coordinating water molecule and the solvent water molecule (O5—H4···O7 and O5—H5···O8) interact, as well as the solvent water molecules with the methanol molecule (O7—H6···O6 and O8—H17···O6). The solvent water molecules are also donors to the free sulfate O atoms of the complex (O7—H7···O4; O8—H18···O2). The complex is also connected *via* its amine function as donor molecule to an the adjacent complex (N5—H5···O2).There is also an intramolecular hydrogen bond in the complex, which involves the amine group and the O atom from sulfate anion (N5—H5A···O4).

π—π interactions between symmetry-related phenantroline moieties with a shortest centroid to centroid interaction of 3.573 (2) Å may consolidate the crystal packing.

### Experimental

A 10 mL methanol solution of 1-(1,10-phenanthrolin-9-yl)-1H-pyrazol-3-amine (0.0549 g, 0.21 mmol) was added into a 10 mL water solution containing CuSO_4_^.^5H_2_O (0.0549 g, 0.22 mmol), and the resulting solution was stirred for a few minutes. Yellow single crystals were obtained after the filtrate had been allowed to stand at room temperature for about one week.

### Refinement

The positions of the H atoms of the water molecule and hydroxyl group were located in a difference map; other H atoms were placed in calculated positions. All H atoms were refined as riding with C—H = 0.96 Å, *U*_iso_ = 1.5*U*_eq_(C) for methyl group; C—H = 0.93 Å, *U*_iso_ = 1.2*U*_eq_(C) for aromatic groups; N—H = 0.86 Å, *U*_iso_ = 1.2*U*_eq_(N); O—H = 0.84-0.90 Å, *U*_iso_ = 1.5*U*_eq_(O).

### Crystal data

**Table:**

[Cu(SO_4_)(C_15_H_11_N_5_)(H_2_O)]·CH_4_O·2H_2_O	*F*(000) = 1044
*M**_r_* = 506.98	*D*_x_ = 1.666 Mg m^−^^3^
Monoclinic, *P*2_1_/*n*	Mo *K*α radiation, λ = 0.71073 Å
Hall symbol: -P 2yn	Cell parameters from 2683 reflections
*a* = 8.0190 (13) Å	θ = 2.7–23.6°
*b* = 18.489 (3) Å	µ = 1.24 mm^−^^1^
*c* = 14.086 (2) Å	*T* = 298 K
β = 104.551 (2)°	Prism, yellow
*V* = 2021.4 (6) Å^3^	0.22 × 0.15 × 0.11 mm
*Z* = 4	

### Data collection

**Table:**

Bruker SMART APEX CCD diffractometer	4394 independent reflections
Radiation source: fine-focus sealed tube	3434 reflections with *I* > 2σ(*I*)
Graphite monochromator	*R*_int_ = 0.038
φ and ω scans	θ_max_ = 27.0°, θ_min_ = 1.9°
Absorption correction: multi-scan (*SADABS*; Sheldrick, 1996)	*h* = −10→7
*T*_min_ = 0.772, *T*_max_ = 0.876	*k* = −23→19
11702 measured reflections	*l* = −17→17

### Refinement

**Table:**

Refinement on *F*^2^	Primary atom site location: structure-invariant direct methods
Least-squares matrix: full	Secondary atom site location: difference Fourier map
*R*[*F*^2^ > 2σ(*F*^2^)] = 0.047	Hydrogen site location: inferred from neighbouring sites
*wR*(*F*^2^) = 0.119	H-atom parameters constrained
*S* = 1.03	*w* = 1/[σ^2^(*F*_o_^2^) + (0.0628*P*)^2^ + 0.2654*P*] where *P* = (*F*_o_^2^ + 2*F*_c_^2^)/3
4394 reflections	(Δ/σ)_max_ = 0.001
281 parameters	Δρ_max_ = 0.52 e Å^−^^3^
0 restraints	Δρ_min_ = −0.31 e Å^−^^3^

### Special details

**Table:**

Geometry. All esds (except the esd in the dihedral angle between two l.s. planes) are estimated using the full covariance matrix. The cell esds are taken into account individually in the estimation of esds in distances, angles and torsion angles; correlations between esds in cell parameters are only used when they are defined by crystal symmetry. An approximate (isotropic) treatment of cell esds is used for estimating esds involving l.s. planes.
Refinement. Refinement of F^2^ against ALL reflections. The weighted R-factor wR and goodness of fit S are based on F^2^, conventional R-factors R are based on F, with F set to zero for negative F^2^. The threshold expression of F^2^ > 2sigma(F^2^) is used only for calculating R-factors(gt) etc. and is not relevant to the choice of reflections for refinement. R-factors based on F^2^ are statistically about twice as large as those based on F, and R- factors based on ALL data will be even larger.

### Fractional atomic coordinates and isotropic or equivalent isotropic displacement parameters (Å^2^)

**Table:**

	*x*	*y*	*z*	*U*_iso_*/*U*_eq_	
C1	0.1583 (4)	0.18352 (19)	−0.0328 (3)	0.0448 (8)	
H1	0.1747	0.2313	−0.0121	0.054*	
C2	0.0344 (5)	0.1677 (2)	−0.1206 (3)	0.0554 (10)	
H2	−0.0272	0.2050	−0.1579	0.066*	
C3	0.0052 (5)	0.0986 (2)	−0.1506 (3)	0.0541 (10)	
H3	−0.0761	0.0883	−0.2088	0.065*	
C4	0.0969 (4)	0.04186 (19)	−0.0944 (2)	0.0423 (8)	
C5	0.2197 (4)	0.06290 (17)	−0.0093 (2)	0.0353 (7)	
C6	0.3202 (4)	0.00945 (16)	0.0501 (2)	0.0352 (7)	
C7	0.3036 (4)	−0.06449 (17)	0.0284 (3)	0.0427 (8)	
C8	0.1765 (5)	−0.0840 (2)	−0.0580 (3)	0.0532 (10)	
H8	0.1609	−0.1326	−0.0749	0.064*	
C9	0.0787 (5)	−0.0343 (2)	−0.1156 (3)	0.0532 (10)	
H9	−0.0036	−0.0495	−0.1710	0.064*	
C10	0.4149 (5)	−0.11097 (18)	0.0962 (3)	0.0506 (9)	
H10	0.4066	−0.1607	0.0860	0.061*	
C11	0.5327 (5)	−0.08488 (17)	0.1751 (3)	0.0475 (9)	
H11	0.6048	−0.1159	0.2189	0.057*	
C12	0.5431 (4)	−0.00930 (16)	0.1890 (2)	0.0362 (7)	
C13	0.7915 (4)	0.00404 (19)	0.3364 (3)	0.0481 (9)	
H13	0.8215	−0.0438	0.3523	0.058*	
C14	0.8710 (5)	0.0619 (2)	0.3822 (3)	0.0493 (9)	
H14	0.9655	0.0622	0.4364	0.059*	
C15	0.7838 (4)	0.12364 (18)	0.3324 (2)	0.0385 (7)	
C31	0.1612 (7)	0.4606 (4)	0.1058 (4)	0.111 (2)	
H31A	0.2316	0.4733	0.1694	0.166*	
H31B	0.0442	0.4747	0.1012	0.166*	
H31C	0.2027	0.4852	0.0563	0.166*	
Cu1	0.44408 (4)	0.138051 (18)	0.15183 (3)	0.03011 (13)	
N1	0.2516 (3)	0.13245 (13)	0.02083 (18)	0.0343 (6)	
N2	0.4373 (3)	0.03481 (13)	0.12916 (18)	0.0334 (6)	
N3	0.6532 (3)	0.10168 (13)	0.25914 (18)	0.0347 (6)	
N4	0.6587 (3)	0.02680 (13)	0.26229 (19)	0.0370 (6)	
N5	0.8209 (4)	0.19272 (15)	0.3524 (2)	0.0529 (8)	
H5A	0.7596	0.2259	0.3174	0.063*	
H5B	0.9063	0.2044	0.4004	0.063*	
O1	0.4992 (3)	0.35921 (11)	0.0990 (2)	0.0515 (6)	
O2	0.6055 (3)	0.25862 (13)	0.02490 (17)	0.0525 (6)	
O3	0.4648 (3)	0.24075 (11)	0.15597 (17)	0.0434 (6)	
O4	0.7439 (3)	0.29447 (14)	0.18900 (18)	0.0538 (7)	
O5	0.2591 (3)	0.13987 (12)	0.24556 (18)	0.0480 (6)	
H4	0.1973	0.1805	0.2347	0.072*	
H5	0.3107	0.1423	0.3096	0.072*	
O6	0.1687 (4)	0.3845 (2)	0.0919 (3)	0.0984 (12)	
H12	0.2611	0.3620	0.0915	0.148*	
O7	0.0713 (4)	0.2581 (2)	0.1844 (3)	0.1175 (16)	
H6	0.1204	0.2972	0.1766	0.176*	
H7	−0.0386	0.2670	0.1828	0.176*	
O8	0.3587 (5)	0.1592 (2)	0.4472 (3)	0.1121 (14)	
H17	0.4504	0.1598	0.4992	0.168*	
H18	0.2726	0.1752	0.4720	0.168*	
S1	0.58095 (10)	0.28883 (4)	0.11578 (6)	0.03254 (19)	

### Atomic displacement parameters (Å^2^)

**Table:**

	*U*^11^	*U*^22^	*U*^33^	*U*^12^	*U*^13^	*U*^23^
C1	0.046 (2)	0.046 (2)	0.0389 (18)	−0.0066 (15)	0.0051 (15)	0.0077 (15)
C2	0.046 (2)	0.077 (3)	0.0372 (19)	−0.0089 (19)	−0.0022 (16)	0.0165 (19)
C3	0.0403 (19)	0.086 (3)	0.0324 (18)	−0.0197 (19)	0.0020 (15)	−0.0022 (19)
C4	0.0389 (18)	0.061 (2)	0.0299 (16)	−0.0185 (16)	0.0142 (14)	−0.0122 (15)
C5	0.0363 (17)	0.0417 (17)	0.0304 (15)	−0.0119 (14)	0.0127 (13)	−0.0075 (13)
C6	0.0352 (16)	0.0366 (16)	0.0381 (17)	−0.0090 (13)	0.0175 (14)	−0.0069 (14)
C7	0.0465 (19)	0.0374 (17)	0.053 (2)	−0.0135 (15)	0.0298 (16)	−0.0170 (16)
C8	0.057 (2)	0.048 (2)	0.064 (3)	−0.0223 (18)	0.032 (2)	−0.0255 (19)
C9	0.050 (2)	0.070 (3)	0.044 (2)	−0.0303 (19)	0.0202 (17)	−0.0283 (19)
C10	0.061 (2)	0.0277 (16)	0.075 (3)	−0.0035 (16)	0.039 (2)	−0.0118 (18)
C11	0.053 (2)	0.0319 (17)	0.064 (2)	0.0059 (15)	0.0256 (18)	0.0052 (16)
C12	0.0389 (17)	0.0307 (15)	0.0439 (18)	0.0049 (13)	0.0199 (15)	0.0023 (14)
C13	0.053 (2)	0.048 (2)	0.043 (2)	0.0213 (17)	0.0115 (17)	0.0141 (17)
C14	0.049 (2)	0.055 (2)	0.0372 (18)	0.0147 (17)	−0.0026 (16)	0.0036 (16)
C15	0.0375 (17)	0.0467 (19)	0.0293 (16)	0.0077 (14)	0.0048 (14)	0.0000 (14)
C31	0.090 (4)	0.147 (6)	0.096 (4)	0.053 (4)	0.026 (3)	0.035 (4)
Cu1	0.0322 (2)	0.0259 (2)	0.0296 (2)	−0.00044 (14)	0.00279 (15)	−0.00061 (14)
N1	0.0318 (13)	0.0393 (14)	0.0302 (13)	−0.0064 (11)	0.0049 (11)	0.0000 (11)
N2	0.0378 (14)	0.0286 (13)	0.0354 (14)	−0.0010 (10)	0.0123 (12)	−0.0030 (11)
N3	0.0386 (15)	0.0293 (13)	0.0341 (14)	0.0055 (11)	0.0049 (11)	0.0012 (11)
N4	0.0407 (15)	0.0326 (14)	0.0376 (14)	0.0116 (11)	0.0095 (12)	0.0035 (11)
N5	0.0575 (19)	0.0450 (17)	0.0422 (17)	0.0028 (14)	−0.0134 (15)	−0.0033 (14)
O1	0.0539 (15)	0.0301 (12)	0.0713 (18)	0.0039 (10)	0.0170 (13)	0.0049 (11)
O2	0.0635 (16)	0.0543 (15)	0.0387 (13)	−0.0005 (12)	0.0110 (12)	−0.0137 (11)
O3	0.0458 (13)	0.0280 (11)	0.0564 (15)	−0.0053 (9)	0.0129 (11)	−0.0011 (10)
O4	0.0359 (13)	0.0714 (17)	0.0474 (15)	−0.0082 (12)	−0.0021 (11)	−0.0075 (12)
O5	0.0465 (14)	0.0536 (15)	0.0445 (14)	0.0042 (11)	0.0128 (11)	−0.0029 (11)
O6	0.054 (2)	0.094 (3)	0.153 (4)	0.0053 (18)	0.037 (2)	0.015 (2)
O7	0.077 (2)	0.103 (3)	0.195 (4)	0.046 (2)	0.075 (3)	0.068 (3)
O8	0.097 (3)	0.176 (4)	0.057 (2)	0.041 (3)	0.0068 (19)	−0.019 (2)
S1	0.0335 (4)	0.0271 (4)	0.0331 (4)	−0.0026 (3)	0.0009 (3)	−0.0041 (3)

### Geometric parameters (Å, º)

**Table:**

C1—N1	1.318 (4)	C14—C15	1.427 (4)
C1—C2	1.408 (5)	C14—H14	0.9300
C1—H1	0.9300	C15—N5	1.325 (4)
C2—C3	1.349 (6)	C15—N3	1.336 (4)
C2—H2	0.9300	C31—O6	1.424 (6)
C3—C4	1.404 (5)	C31—H31A	0.9600
C3—H3	0.9300	C31—H31B	0.9600
C4—C5	1.402 (4)	C31—H31C	0.9600
C4—C9	1.439 (5)	Cu1—O3	1.906 (2)
C5—N1	1.358 (4)	Cu1—N2	1.934 (2)
C5—C6	1.410 (4)	Cu1—N3	2.068 (2)
C6—N2	1.348 (4)	Cu1—N1	2.090 (2)
C6—C7	1.400 (4)	Cu1—O5	2.220 (2)
C7—C10	1.421 (5)	N3—N4	1.386 (3)
C7—C8	1.424 (5)	N5—H5A	0.8600
C8—C9	1.342 (5)	N5—H5B	0.8600
C8—H8	0.9300	O1—S1	1.450 (2)
C9—H9	0.9300	O2—S1	1.455 (2)
C10—C11	1.354 (5)	O3—S1	1.498 (2)
C10—H10	0.9300	O4—S1	1.451 (2)
C11—C12	1.411 (4)	O5—H4	0.8913
C11—H11	0.9300	O5—H5	0.8938
C12—N2	1.317 (4)	O6—H12	0.8515
C12—N4	1.375 (4)	O7—H6	0.8442
C13—C14	1.328 (5)	O7—H7	0.8906
C13—N4	1.357 (4)	O8—H17	0.8981
C13—H13	0.9300	O8—H18	0.8983
			
N1—C1—C2	121.9 (3)	O6—C31—H31A	109.5
N1—C1—H1	119.1	O6—C31—H31B	109.5
C2—C1—H1	119.1	H31A—C31—H31B	109.5
C3—C2—C1	120.0 (4)	O6—C31—H31C	109.5
C3—C2—H2	120.0	H31A—C31—H31C	109.5
C1—C2—H2	120.0	H31B—C31—H31C	109.5
C2—C3—C4	120.4 (3)	O3—Cu1—N2	170.82 (10)
C2—C3—H3	119.8	O3—Cu1—N3	104.67 (10)
C4—C3—H3	119.8	N2—Cu1—N3	77.52 (10)
C5—C4—C3	115.5 (3)	O3—Cu1—N1	96.64 (10)
C5—C4—C9	117.5 (3)	N2—Cu1—N1	79.71 (10)
C3—C4—C9	127.0 (3)	N3—Cu1—N1	155.91 (10)
N1—C5—C4	124.5 (3)	O3—Cu1—O5	91.88 (9)
N1—C5—C6	116.3 (3)	N2—Cu1—O5	96.76 (9)
C4—C5—C6	119.2 (3)	N3—Cu1—O5	96.34 (10)
N2—C6—C7	121.9 (3)	N1—Cu1—O5	94.02 (10)
N2—C6—C5	115.0 (3)	C1—N1—C5	117.6 (3)
C7—C6—C5	123.1 (3)	C1—N1—Cu1	131.1 (2)
C6—C7—C10	115.8 (3)	C5—N1—Cu1	111.23 (19)
C6—C7—C8	116.3 (3)	C12—N2—C6	121.1 (3)
C10—C7—C8	127.9 (3)	C12—N2—Cu1	121.2 (2)
C9—C8—C7	121.8 (3)	C6—N2—Cu1	117.7 (2)
C9—C8—H8	119.1	C15—N3—N4	105.4 (2)
C7—C8—H8	119.1	C15—N3—Cu1	143.2 (2)
C8—C9—C4	122.1 (3)	N4—N3—Cu1	111.22 (18)
C8—C9—H9	118.9	C13—N4—C12	132.7 (3)
C4—C9—H9	118.9	C13—N4—N3	110.3 (3)
C11—C10—C7	121.8 (3)	C12—N4—N3	116.8 (2)
C11—C10—H10	119.1	C15—N5—H5A	120.0
C7—C10—H10	119.1	C15—N5—H5B	120.0
C10—C11—C12	118.1 (3)	H5A—N5—H5B	120.0
C10—C11—H11	120.9	S1—O3—Cu1	129.60 (14)
C12—C11—H11	120.9	Cu1—O5—H4	109.7
N2—C12—N4	112.6 (3)	Cu1—O5—H5	113.0
N2—C12—C11	121.2 (3)	H4—O5—H5	103.1
N4—C12—C11	126.2 (3)	C31—O6—H12	123.5
C14—C13—N4	108.2 (3)	H6—O7—H7	109.3
C14—C13—H13	125.9	H17—O8—H18	103.3
N4—C13—H13	125.9	O1—S1—O4	109.94 (15)
C13—C14—C15	106.8 (3)	O1—S1—O2	110.92 (15)
C13—C14—H14	126.6	O4—S1—O2	110.86 (15)
C15—C14—H14	126.6	O1—S1—O3	107.19 (14)
N5—C15—N3	123.2 (3)	O4—S1—O3	107.99 (14)
N5—C15—C14	127.6 (3)	O2—S1—O3	109.84 (14)
N3—C15—C14	109.2 (3)		
			
N1—C1—C2—C3	1.8 (6)	N4—C12—N2—C6	176.8 (3)
C1—C2—C3—C4	0.4 (6)	C11—C12—N2—C6	−2.9 (4)
C2—C3—C4—C5	−1.7 (5)	N4—C12—N2—Cu1	−3.3 (4)
C2—C3—C4—C9	179.0 (3)	C11—C12—N2—Cu1	177.0 (2)
C3—C4—C5—N1	0.9 (5)	C7—C6—N2—C12	0.9 (4)
C9—C4—C5—N1	−179.7 (3)	C5—C6—N2—C12	−177.8 (3)
C3—C4—C5—C6	−178.5 (3)	C7—C6—N2—Cu1	−179.0 (2)
C9—C4—C5—C6	0.9 (4)	C5—C6—N2—Cu1	2.3 (3)
N1—C5—C6—N2	−0.8 (4)	N3—Cu1—N2—C12	5.9 (2)
C4—C5—C6—N2	178.7 (3)	N1—Cu1—N2—C12	178.0 (2)
N1—C5—C6—C7	−179.5 (3)	O5—Cu1—N2—C12	−89.1 (2)
C4—C5—C6—C7	−0.1 (5)	N3—Cu1—N2—C6	−174.2 (2)
N2—C6—C7—C10	1.4 (4)	N1—Cu1—N2—C6	−2.1 (2)
C5—C6—C7—C10	−179.9 (3)	O5—Cu1—N2—C6	90.8 (2)
N2—C6—C7—C8	−179.2 (3)	N5—C15—N3—N4	179.5 (3)
C5—C6—C7—C8	−0.5 (5)	C14—C15—N3—N4	−0.4 (3)
C6—C7—C8—C9	0.3 (5)	N5—C15—N3—Cu1	−5.7 (6)
C10—C7—C8—C9	179.6 (3)	C14—C15—N3—Cu1	174.4 (3)
C7—C8—C9—C4	0.6 (5)	O3—Cu1—N3—C15	7.6 (4)
C5—C4—C9—C8	−1.2 (5)	N2—Cu1—N3—C15	178.4 (4)
C3—C4—C9—C8	178.2 (3)	N1—Cu1—N3—C15	159.1 (3)
C6—C7—C10—C11	−1.8 (5)	O5—Cu1—N3—C15	−86.0 (4)
C8—C7—C10—C11	178.9 (3)	O3—Cu1—N3—N4	−177.80 (18)
C7—C10—C11—C12	0.0 (5)	N2—Cu1—N3—N4	−6.98 (18)
C10—C11—C12—N2	2.5 (5)	N1—Cu1—N3—N4	−26.3 (4)
C10—C11—C12—N4	−177.3 (3)	O5—Cu1—N3—N4	88.58 (19)
N4—C13—C14—C15	−1.0 (4)	C14—C13—N4—C12	175.1 (3)
C13—C14—C15—N5	−179.0 (3)	C14—C13—N4—N3	0.8 (4)
C13—C14—C15—N3	0.8 (4)	N2—C12—N4—C13	−177.5 (3)
C2—C1—N1—C5	−2.6 (5)	C11—C12—N4—C13	2.2 (6)
C2—C1—N1—Cu1	179.4 (3)	N2—C12—N4—N3	−3.4 (4)
C4—C5—N1—C1	1.2 (5)	C11—C12—N4—N3	176.3 (3)
C6—C5—N1—C1	−179.3 (3)	C15—N3—N4—C13	−0.2 (3)
C4—C5—N1—Cu1	179.7 (2)	Cu1—N3—N4—C13	−176.9 (2)
C6—C5—N1—Cu1	−0.9 (3)	C15—N3—N4—C12	−175.6 (3)
O3—Cu1—N1—C1	−8.8 (3)	Cu1—N3—N4—C12	7.8 (3)
N2—Cu1—N1—C1	179.8 (3)	N3—Cu1—O3—S1	77.66 (19)
N3—Cu1—N1—C1	−161.0 (3)	N1—Cu1—O3—S1	−91.02 (19)
O5—Cu1—N1—C1	83.6 (3)	O5—Cu1—O3—S1	174.72 (18)
O3—Cu1—N1—C5	173.1 (2)	Cu1—O3—S1—O1	155.30 (18)
N2—Cu1—N1—C5	1.6 (2)	Cu1—O3—S1—O4	−86.3 (2)
N3—Cu1—N1—C5	20.8 (4)	Cu1—O3—S1—O2	34.7 (2)
O5—Cu1—N1—C5	−94.6 (2)		

### Hydrogen-bond geometry (Å, º)

**Table:**

*D*—H···*A*	*D*—H	H···*A*	*D*···*A*	*D*—H···*A*
O5—H4···O7	0.89	1.79	2.671 (4)	167
O5—H5···O8	0.89	1.90	2.774 (4)	164
O6—H12···O1	0.85	1.89	2.668 (4)	152
O7—H6···O6	0.84	2.10	2.878 (5)	153
O7—H7···O4^i^	0.89	1.84	2.726 (4)	173
O8—H17···O6^ii^	0.90	2.07	2.906 (5)	154
O8—H18···O2^iii^	0.90	2.09	2.957 (4)	163
N5—H5*A*···O4	0.86	2.19	2.916 (4)	143
N5—H5*B*···O2^ii^	0.86	2.17	3.022 (4)	175

Symmetry codes: (i) *x*−1, *y*, *z*; (ii) *x*+1/2, −*y*+1/2, *z*+1/2; (iii) *x*−1/2, −*y*+1/2, *z*+1/2.
